# Evaluation of furosemide regimens in neonates treated with extracorporeal membrane oxygenation

**DOI:** 10.1186/cc5115

**Published:** 2006-12-01

**Authors:** Maria MJ van der Vorst, Enno Wildschut, Robbert J Houmes, Saskia J Gischler, Joana E Kist-van Holthe, Jacobus Burggraaf, Albert J van der Heijden, Dick Tibboel

**Affiliations:** 1 Department of Paediatrics, University of Kuwait, P.O. Box 24923, Safat 13110, Kuwait; 2 Department of Paediatric Surgery, Erasmus MC, Sophia Children's Hospital, Dr Molewaterplein 60, 3000 CB Rotterdam, The Netherlands; 3 Department of Paediatrics, Leiden University Medical Centre, Albinusdreef 2, 2300 RC Leiden, The Netherlands; 4 Centre for Human Drug Research, Zernikedreef 10, 2333 CL Leiden, The Netherlands; 5 Department of Paediatrics, Erasmus MC, Sophia Children's Hospital, Dr Molewaterplein 60, 3000 CB Rotterdam, The Netherlands

## Abstract

**Introduction:**

Loop diuretics are the most frequently used diuretics in patients treated with extracorporeal membrane oxygenation (ECMO). In patients after cardiopulmonary bypass (CPB) surgery, the use of continuous furosemide infusion is increasingly documented. Because ECMO and CPB are 'comparable' procedures, continuous furosemide infusion is used in newborns on ECMO. We report on the use of continuous intravenous furosemide in neonates treated with ECMO.

**Methods:**

This was a retrospective observational study in neonates treated with continuous intravenous furosemide during ECMO.

**Results:**

Thirty-one patients were included in the study. A median of 25 (9–149) hours after the start of ECMO, continuous furosemide therapy was started at a median rate of 0.08 (0.02–0.17) mg/kg per hour. The continuous furosemide dose was not changed in the individual patient. Seven patients received a furosemide bolus prior to, and five patients received additional loop diuretics during, the continuous infusion. Urine production before continuous furosemide therapy was not significantly different between patients who received a furosemide bolus prior to the infusion and those who did not receive this bolus (*P *= 0.2879). Although a positive effect of the 'loading' bolus was observed in urine output in the first 24 hours, there was no statistically significant difference in urine output (*P *= 0.0961) or in time (*P *= 0.1976) to reach a urine output of 6 ml/kg per hour between patients. After 24 hours, urine production remained a median of 6.2 ml/kg per hour irrespective of furosemide boluses. The forced diuresis was well tolerated as illustrated by stable haemodynamic parameters and a decrease in ECMO flow and vasopressor score over the observation period.

**Conclusion:**

This is the first report on continuous intravenous furosemide therapy in newborns treated with ECMO. The furosemide regimens used in this study varied widely in continuous and intermittent doses. However, all regimens achieved adequate urine output. An advantage of continuous, over intermittent, intravenous furosemide could not be documented. Furosemide dosing regimens should be developed for neonates treated with ECMO. In addition, therapeutic drug-monitoring studies are required to prevent furosemide toxicity because so far no data are available on serum furosemide levels in neonates treated with ECMO.

## Introduction

Extracorporeal membrane oxygenation (ECMO) is performed in newborns for a variety of diagnoses, including meconium aspiration syndrome (MAS), congenital diaphragmatic hernia (CDH), persistent pulmonary hypertension of the newborn (PPHN), and sepsis/pneumonia [1]. The ECMO circuit, like the cardiopulmonary bypass (CPB) circuit, triggers an important inflammatory reaction and is clinically associated with the so-called capillary leakage syndrome, resulting in intravascular hypovolaemia and renal hypoperfusion [2]. Hence, the ECMO patient usually becomes increasingly oedematous in the initial phase and diuretics are often used to enhance the diuresis to mobilise the fluid excess. Loop diuretics, generally given as an intravenous (IV) bolus, are the most frequently used diuretics in patients treated with ECMO [3]. Since the observation that continuous IV furosemide might be superior (especially in haemodynamically unstable patients) to intermittent administration in infants after cardiac surgery, the use of continuous furosemide infusion has been increasingly documented in patients after CPB surgery [4-8]. Although there are no data available evaluating the use of continuous IV furosemide in newborns during venoarterial (VA) ECMO, continuous furosemide infusion is used increasingly in our unit in newborns treated with ECMO because ECMO and CPB are 'comparable' procedures. Although the dosing schedule is largely empirical in this group of patients with varying renal function and altered pharmacokinetics (PK), the current practice is to start with a low furosemide infusion rate (0.05–0.1 mg/kg per hour) [3,9]. We retrospectively studied the use of continuous IV furosemide in neonates treated with VA ECMO over a two year period. In addition, neonates who did not receive continuous IV furosemide during VA ECMO were evaluated.

## Materials and methods

The study was performed at the paediatric surgical intensive care unit (ICU) of the Sophia Children's Hospital of Erasmus Medical Centre in Rotterdam, The Netherlands. This ICU serves as one of two designated ECMO centres in The Netherlands. The medical records of all neonates, who received ECMO treatment between October 2002 and October 2004, were screened for the use of furosemide, continuous and/or intermittent IV, during ECMO treatment and consequently studied by means of chart review in combination with data available in the electronic patient data management system.

Demographic and clinical data recorded included gestational and postpartum age, gender, weight, diagnosis, ECMO flow and duration of ECMO treatment, time (after starting ECMO) continuous furosemide infusion was started, dose and duration of continuous IV furosemide, additional loop diuretics, inotropic support, and fluid intake. The following variables were measured before and at regular time intervals during the study for a maximum of 72 hours: urine output, heart rate (HR), mean arterial blood pressure, and serum albumin, creatinine, and urea levels.

Continuous IV furosemide was started at the time the patient was cardiovascularly stable. The patient was considered cardiovascularly stable if there was no need for ongoing fluid resuscitation and/or increase in inotropic support. The amount of inotropic support was measured by the vasopressor score [10,11]. During continuous IV furosemide therapy, serum electrolyte levels (sodium, potassium, calcium, and magnesium) were closely monitored and supplements were given if necessary.

### Statistical analysis

All data are presented as median (range) unless indicated otherwise. Wilcoxon two-sample tests were used for comparison between the different furosemide regimens.

## Results

### General

**Figure 1:**
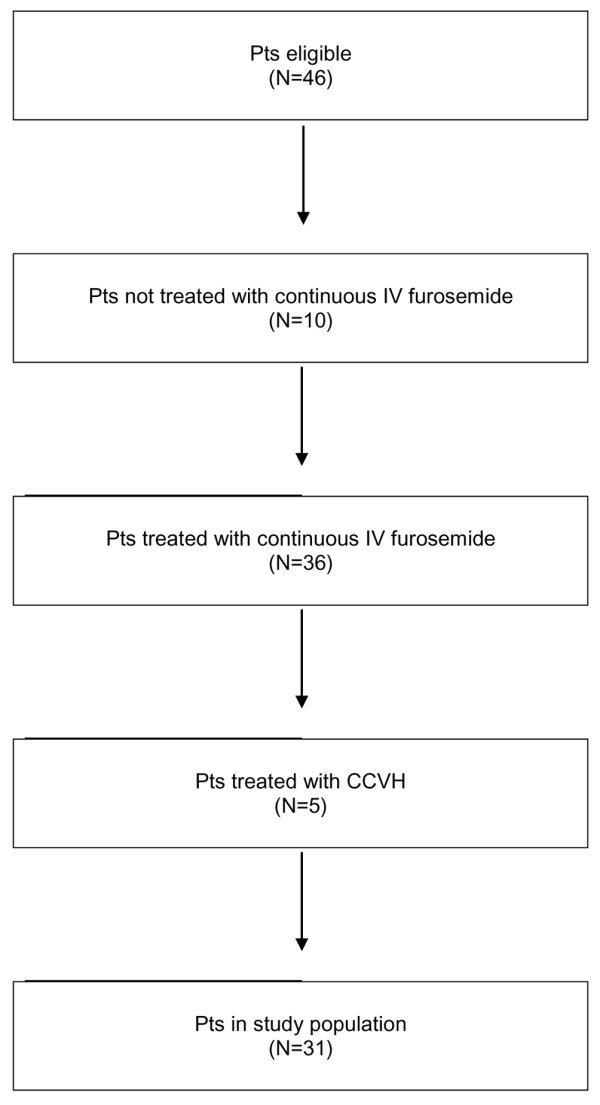
Flowchart of patient recruitment. CCVH, continuous venovenous haemofiltration; IV, intravenous; Pts, patients.

Forty-six patients in whom VA ECMO was performed were eligible for the study. Ten patients were excluded from the study because they did not receive continuous IV furosemide during ECMO. Thirty-six patients were enrolled in the study. Five patients were excluded from analysis because they were treated with continuous veno-arterial haemofiltration (CAVH). Three patients were treated with CAVH because of acute renal failure (median creatinine 90 μmol/l and urea 22.7 mmol/l) and two patients were treated from the start of ECMO with CAVH (trial). Thirty-one patients were analysed (Figure 1). The study population consisted of 12 female and 19 male patients. Median gestational age was 40 (35–43) weeks. On admission, median postpartum age was 1 (0–16) days and median weight was 3.5 (2.3–5.2) kg. ECMO was performed for MAS in 10 patients, for CDH in 13 patients, for sepsis/pneumonia in five patients, for PPHN in two patients, and for cardiomyopathy in one patient. ECMO was started a median of 4 (0–46) hours after admission. All patients were weaned from ECMO after a median of 127 (44–339) hours. The median stay in the ICU was 11 (3–186) days. Due to recurrent and therapy-resistant pulmonary hypertension, five patients with CDH died before discharge from the ICU.

### Furosemide regimen

Prior to the start of continuous IV furosemide, seven patients received an IV furosemide bolus (dose 1 [0.4–2.4] mg/kg). Continuous IV furosemide therapy was started a median of 25 (9–149) hours after the start of ECMO at a median rate of 0.08 (0.02–0.17) mg/kg per hour. The continuous furosemide therapy in patients with CDH was started after a median of 33 (11–149) hours. The continuous furosemide dose in the patients who received a bolus prior to the infusion was 0.08 (0.04–0.13) mg/kg per hour; in the patients who did not receive a bolus, the dose was 0.08 (0.02–0.17) mg/kg per hour. The furosemide dose was not changed in the individual patient during the study period. The administered continuous IV furosemide dose over the span of 24 hours was a median of 1.92 (0.48–4.08) mg/kg.

During the study period, five patients received additional loop diuretics: four patients received a total median furosemide dose of 7 (5.6–10.8) mg/kg, and one received a total bumetanide dose of 0.1 mg/kg. The total administered continuous and intermittent IV furosemide doses on the first, second, and third days of the study were 1.92 (0.48–6.6), 1.92 (0.96–6.6), and 2.0 (0.5–6.6) mg/kg per 24 hours, respectively. The furosemide regimen is depicted in Table 1.

In 10 patients, continuous furosemide infusion was discontinued a median of 2 (0–144) hours before decannulation, and in 21 patients it was discontinued a median of 25 (4–623) hours after decannulation. The duration of the continuous furosemide infusion during ECMO was a median of 98 (21–294) hours, which is in accordance with a median of 80% (29%–95%) of the ECMO time.

### Furosemide effects

**Figure 2:**
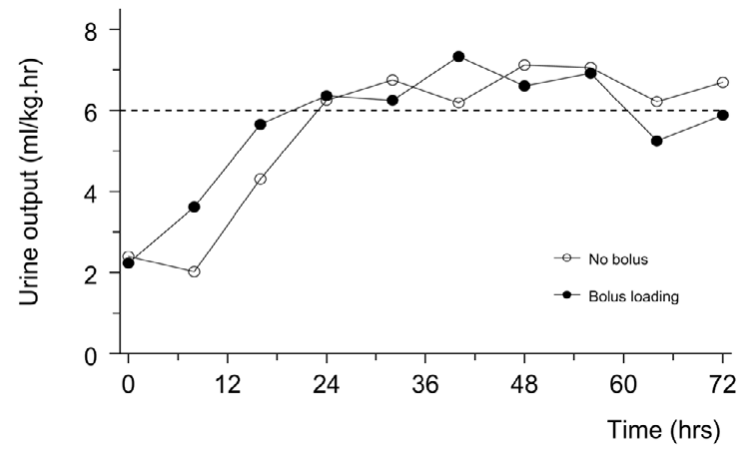
Median urine production over the observation period. The line with closed circles depicts the median urine production of the patients (*n *= 7) who received a furosemide bolus prior to the continuous infusion. The line with open circles depicts the median urine production of the patients (*n *= 24) who did not receive a furosemide bolus prior to the continuous infusion.

In the patients (*n *= 7) who received a furosemide bolus prior to the continuous infusion, median urine production before the start of continuous infusion was 2.2 ml/kg per hour; in the patients (*n *= 24) who did not receive this furosemide bolus, it was 2.4 ml/kg per hour (*P *= 0.2879). Median urine production increased to 3.6, 5.7, and 6.4 ml/kg per hour, respectively, after 8, 16, and 24 hours of furosemide infusion in the patients (*n *= 7) who received a furosemide bolus prior to the continuous infusion; in the patients (*n *= 24) who did not receive a furosemide bolus, urine production values were 2.0, 4.3, and 6.3 ml/kg per hour, respectively (*P *= 0.0961). The time that urine production of 6 ml/kg per hour was reached in the patients with and without a bolus prior to the continuous infusion was not significantly different (*P *= 0.1976). Median urine production remained 6.2 ml/kg per hour after 24 hours of continuous furosemide infusion in all patients irrespective of a bolus prior to the continuous furosemide infusion. Urine production is shown in Figure 2.

Fluid balances, calculated over eight hour intervals, were a median of +79.4 ml before the start of continuous furosemide infusion in the patients who received a furosemide bolus prior and +98.0 ml in the patients who did not receive this bolus. Median fluid balances in the patients who received a furosemide bolus prior were +76.9, -21, and -10.5 ml, respectively, after 8, 16, and 24 hours of continuous furosemide therapy. In the patients who did not receive a furosemide bolus prior to the furosemide infusion, the median fluid balances after 8, 16, and 24 hours of continuous furosemide therapy were +106.4, +28.2, and +12.0 ml, respectively.

### ECMO regimen

The priming volume of the ECMO circuit was approximately 400 ml, the solution consisted of albumin and packed red blood cells, and the initial median ECMO flow was 130 (82–185) ml/kg per minute, equalling 80% of the total cardiac output. Median ECMO flow values at the start of the continuous furosemide and after 8, 24, 48, and 72 hours of continuous furosemide were 87 (31–147), 86 (15–144), 76 (13–153), 50 (14–95), and 59 (14–90) ml/kg per minute, respectively. The ECMO flow in the CDH patients was not significantly different.

### Cardiovascular effects

Median mean arterial pressure (MAP) and HR at the start of ECMO and at the start of the furosemide treatment were 50 (38–78) mm Hg and 167 (102–237) beats per minute and 51 (37–74) mm Hg and 138 (88–198) beats per minute, respectively. Median MAP and HR after 8, 24, 48, and 72 hours of furosemide treatment were 52 (38–72) mm Hg and 134 (109–171) beats per minute, 52 (37–127) mm Hg and 140 (107–185) beats per minute, 54 (40–80) mm Hg and 143 (94–196) beats per minute, and 51 (40–65) mm Hg and 145 (98–189) beats per minute, respectively. All cardiovascular parameters were within the normal range for age [12,13]. All patients remained cardiovascularly stable during the administration of continuous IV furosemide and the inotropic support was gradually decreased during the observation period as illustrated by the vasopressor score. The number of patients requiring inotropic support during the study was decreased from 25/31 (81%) to 16/31 (52%). Median vasopressor scores at the start of ECMO and at the start of the continuous furosemide infusion were 11 (0–196) and 5 (0–170), respectively. Median vasopressor scores after 8, 24, 48, and 72 hours of continuous furosemide were 5 (0–170), 5 (0–170), 5 (0–170), and 5 (0–30), respectively. Inotropic support was significantly higher in the CDH patients. Median vasopressor scores of the CDH patients at the start of ECMO, at the start of continuous furosemide infusion, and after 8, 24, 48, and 72 hours of continuous furosemide infusion were 33 (0–170), 20 (0–170), 20 (0–170), 20 (0–170), 17 (0–170), and 12.5 (0–30), respectively.

### Renal function

Median serum creatinine levels at the start of ECMO and at the start of continuous IV furosemide infusion were 55 (14–90) and 52 (14–90) μmol/l, respectively. Median serum creatinine levels after 24, 48, and 72 hours of continuous IV furosemide treatment were 50 (19–79), 49 (20–79), and 43 (22–66) μmol/l, respectively. Median serum urea levels at the start of ECMO and at the start of continuous IV furosemide were 3.1 (1–9.7) and 2.8 (1.3–6.5) mmol/l, respectively. After 24, 48, and 72 hours of furosemide infusion, median serum urea levels were 4.0 (1.5–23), 4.4 (1.5–8.6), and 5.4 (1.3–11.6) mmol/l, respectively. Median serum albumin levels at the start of ECMO and at the start of furosemide infusion were 16 (4–27) and 27 (16–36) g/l, respectively. During continuous IV furosemide treatment, median serum albumin levels were 27 (21–36), 29 (16–41), and 30 (24–40) g/l after 24, 48, and 72 hours, respectively.

### Patients who did not receive continuous IV furosemide during VA ECMO

#### General

Ten patients did not receive continuous IV furosemide during ECMO. Two patients were excluded from this evaluation because they were treated with CAVH. One patient was treated with CAVH because of acute renal failure (creatinine 74 μmol/l and urea 4.8 mmol/l) and the other patient was treated from the start of ECMO with CAVH (trial). Eight patients were evaluated. This group consisted of five female and three male patients. Median gestational age was 40 (36–42) weeks. On admission, median postpartum age was 1 (0–6) days and median weight was 3.3 (1.9–3.7) kg. ECMO was performed for MAS in three patients, for CDH in two patients, for sepsis in two patients, and in one patient for pulmonary hypertension after pneumonectomy due to a congenital cystic adenomatoid malformation of the lung. ECMO was started a median of 0 (0–198) hours after admission. Seven patients were weaned from ECMO after a median of 98 (8–275) hours. The median stay in the ICU was 6 (0–22) days. One patient with sepsis died on ECMO.

#### Furosemide regimen

Only three patients received intermittent IV furosemide. One patient received the first bolus 32 hours before the start of ECMO, and the other two patients started with intermittent IV furosemide 18 and 159 hours, respectively, after the start of ECMO. The furosemide doses before ECMO and on the first, second, and third days after the start of ECMO were 1.84, 1, 5, and 5 mg/kg per 24 hours, respectively, and 1 mg/kg per 24 hours in the patient who started furosemide after 159 hours on ECMO.

#### Urine production and fluid balance

Median urine production values after 24, 48, and 72 hours on ECMO were 4.4, 5.4, and 5.6 ml/kg per hour, respectively. Median fluid balances after 24, 48, and 72 hours on ECMO were +173, +34, and +11.9 ml, respectively.

#### ECMO regimen

The priming volume of the ECMO circuit was approximately 400 ml, the solution consisted of albumin and packed red blood cells, and the initial median ECMO flow was 146 (111–161) ml/kg per minute, equalling 80% of the total cardiac output. Median ECMO flow values after 24, 48, and 72 hours on ECMO were 135 (56–189), 116 (80–126), and 116 (80–126) ml/kg minute, respectively.

#### Cardiovascular effects

Median MAP and HR at the start of ECMO and after 24, 48, and 72 hours on ECMO were 45 (30–79) mm Hg and 148 (112–291) beats per minute, 48 (43–56) mm Hg and 146 (93–171) beats per minute, 47 (42–55) mm Hg and 130 (107–162) beats per minute, and 51 (48–56) mm Hg and 124 (114–180) beats per minute, respectively. At the start of ECMO and after 24, 48, and 72 hours on ECMO, eight, five, four, and four patients, respectively, received inotropic support. Median vasopressor scores at the start of ECMO and after 24, 48, and 72 hours on ECMO were 23 (2–85), 5 (0–42), 3 (0–40), and 5 (0–40), respectively.

#### Renal function

Median serum creatinine levels at the start of ECMO and after 24, 48, and 72 hours on ECMO were 47 (21–121), 45 (24–55), 47 (24–87), and 38 (25–85) μmol/l, respectively. Median serum urea levels at the start of ECMO and after 24, 48, and 72 hours on ECMO were 2.9 (0.9–10.0), 2.3 (0.9–9.3), 2.4 (1.5–8.5), and 3.5 (1.7–6.5) mmol/l, respectively. Median serum albumin levels at the start of ECMO and after 24, 48, and 72 hours on ECMO were 24 (21–35), 27 (24–30), 28 (26–30), and 27 (24–32) g/l, respectively.

## Discussion

Diuretics, especially loop diuretics, are the mainstay in the enhancement of diuresis in patients treated with ECMO. In contrast to the extensive pharmacokinetic/pharmacodynamic (PK/PD) research on (loop) diuretics in preterm and term neonates, very limited research has been performed on (loop) diuretics in neonates treated with ECMO [3,14]. Wells and colleagues [3] studied the PK/PD of bumetanide in 11 term neonates treated with ECMO and reported that the steady-state volume of distribution and the elimination half-life were greater than comparable values reported in previous studies of bumetanide disposition in premature and term neonates without ECMO and that the plasma clearance was similar for both groups. Although significant diuresis, natriuresis, and kaliuresis were observed with 0.1 mg/kg, the duration of the effects was less than expected given by the prolonged renal elimination.

Since the observation that continuous IV furosemide might be superior (especially in haemodynamically unstable patients) to intermittent administration in infants and children after CPB surgery, continuous furosemide infusions have been increasingly used in patients after cardiac surgery [4-7]. Trials assessing efficacy and safety of continuous versus intermittent IV furosemide in paediatric patients after CPB surgery revealed that the total furosemide dose administered by continuous infusion was generally less than the dose by intermittent administration [5-8]. No significant difference was observed in the main pharmacodynamic outcome parameter: urine production. However, significantly less variance in urine output was observed in the patients who received a continuous infusion (overview in Table 2). Studies in critically ill adult patients also showed that there was no difference in urine production with continuous IV versus intermittent IV furosemide administration. However, the diuresis was more controlled with fewer haemodynamic and electrolyte variations during continuous furosemide infusion [4,15-18].

Because ECMO and CPB are 'comparable' procedures, continuous furosemide infusion is increasingly used in newborns treated with ECMO. In our unit, continuous IV furosemide therapy was used in 78% of the neonates treated with ECMO. The dosing schedule of continuous IV furosemide in neonates treated with ECMO is largely empirical because of the variable renal function and altered PK [3,9]. This is supported by our observation that the continuous IV furosemide dose varied widely, from 0.02 to 0.17 mg/kg per hour, and that 12/31 (39%) patients received additional loop diuretics. Although the urine output was satisfactory in the patients studied, the use of additional loop diuretics suggests that the applied infusion rates were not optimal. Therefore, dosing regimens for continuous IV furosemide therapy in infants treated with ECMO should be developed. Because ECMO and CPB are 'comparable' procedures, the developed PK/PD model for infants after cardiac surgery might also be applicable for patients treated with ECMO [8,19].

To obtain an acceptable fluid balance (approximately zero) with maintenance fluid of 120 to 140 ml/kg per 24 hours, the target urine production is set at 6 ml/kg per hour in our institution. In all patients studied, the desirable urine output of approximately 6 ml/kg per hour was achieved within 24 hours of continuous IV furosemide infusion and remained at the desired level thereafter, but the furosemide regimens used in our study varied widely. The increased urine production was not correlated with the ECMO flow and the vasopressor score while both were reduced during the observation period. Due to the retrospective nature of our observational study, data on urinary furosemide and sodium excretion were not routinely available to differentiate between increased urine production by furosemide therapy or by clinical improvement.

All patients received continuous IV furosemide at a median rate of 0.08 (0.02–0.17) mg/kg per hour, and 12 patients received additional loop diuretics prior to and/or during the continuous infusion. This illustrates that different regimens are used in the same group of patients and produced similar urinary output. This is in line with the observation in patients after CPB surgery with intermittent versus continuous administration of furosemide [5-7]. In the patients who received a 'loading' bolus, a positive effect was observed in urine output (Figure 2), but no statistically significant difference was reached in urine output in the first 24 hours or in the time to reach a urine output of 6 ml/kg per hour, which might be explained by the inter-individual variability and the difference in group size. In previous studies by our group on infants after CPB surgery, we suggested that continuous IV furosemide therapy would be more effective if initially started at a relatively high infusion rate and preferably preceded by a loading bolus [8,19]. With the developed PK/PD model for infants after cardiac surgery, we simulated various furosemide regimens and observed the effect of a furosemide loading bolus on urine production as well as on the time to reach the predefined urine output [8,19].

The enhanced diuresis was well tolerated as illustrated by the stable haemodynamic parameters and a decrease in ECMO flow and vasopressor score over the observation period. Moreover, the number of patients requiring inotropic support decreased during the study period.

Renal function of the patients studied was within the normal range for age (that is, there were no signs of pre-renal failure before or during furosemide treatment). The observed increase in serum urea levels is most likely due the extremely high rates of whole-body protein breakdown observed in critically ill infants on ECMO [20,21].

The total administered furosemide dose, continuous and intermittent, was a median of 1.92 mg/kg per 24 hours in our study population. This dose is relatively low compared with the continuous IV furosemide dose used in infants and children after CPB surgery [5-8]. In infants after CPB surgery, who received continuous IV furosemide at a rate of 9.6 mg/kg per 24 hours, no toxic serum furosemide levels (>50 μg/ml) were observed [8,22]. A drawback of our retrospective observational study is that serum furosemide levels were not routinely recorded to monitor furosemide toxicity. Because all patients are less than five years of age, we have no routine audiography data. Audiography is performed at the age of five years according to the nationwide standardised evaluation of ECMO patients in The Netherlands to evaluate hearing loss as a sign of furosemide toxicity (among other causes) [23]. An indirect proof of the absence of hearing loss in our patients is the absence of significant delays in language development evaluated at the age of one and two years. Moreover, in the literature, no data are available on serum furosemide levels in newborns treated with ECMO [8]. Therefore, therapeutic drug-monitoring studies are now performed in our centre to prevent furosemide toxicity.

Unfortunately, we could not demonstrate the advantage of continuous IV furosemide over intermittent IV furosemide in our patients. Only eight patients who did not receive continuous IV furosemide were eligible for comparison. Urine production of these patients was a median of 4.4 ml/kg per hour after 24 hours on ECMO, approximately the median time that continuous IV furosemide was started in the study population. Because their diuresis was considered sufficient, (continuous) furosemide therapy was not started.

## Conclusion

To the best of our knowledge, this is the first report on continuous IV furosemide in neonates treated with ECMO and it shows that continuous IV furosemide is frequently used. However, the furosemide regimens used in this study varied widely in continuous and additional intermittent doses. All regimens achieved adequate urine output within 24 hours and no statistically significant difference was observed after a loading bolus. The patients tolerated the forced diuresis well and no adverse effects were observed. However, furosemide toxicity was not evaluated as part of this protocol.

Although the urine output was satisfactory, the furosemide regimens used in this study might not be optimal regimens for newborns treated with ECMO and therefore dosing regimens should be developed. For obvious reasons, our retrospective observational study will not answer the question of whether continuous IV furosemide is the preferred way of administration of furosemide in neonates treated with ECMO.

Currently, a prospective study is being conducted in our unit to evaluate a continuous furosemide regimen, 0.2 mg/kg per hour, based on the PK/PD model developed for infants after CPB surgery for a predefined urine output of approximately 6 ml/kg per hour [19]. During the continuous furosemide infusion, serum furosemide levels are monitored at regular intervals to evaluate furosemide toxicity in newborns treated with ECMO.

## Key messages

• Furosemide regimens in neonates treated with ECMO varied widely in continuous and intermittent doses.

• An advantage of continuous, over intermittent, IV furosemide could not be documented.

## Abbreviations

CAVH = continuous veno-arterial haemofiltration; CDH = congenital diaphragmatic hernia; CPB = cardiopulmonary bypass; ECMO = extracorporeal membrane oxygenation; HR = heart rate; ICU = intensive care unit; IV = intravenous; MAP = mean arterial pressure; MAS = meconium aspiration syndrome; PK = pharmacokinetics; PK/PD = pharmacokinetic/pharmacodynamic; PPHN = persistent pulmonary hypertension of the newborn; VA = venoarterial.

## Competing interests

The authors declare that they have no competing interests.

## Authors' contributions

MvdV evaluated the data and wrote the manuscript. EW, RH, and SG were involved with patient management. JK, JB, and AvdH helped draft the manuscript. DT coordinated the data evaluation and the writing of the manuscript. All authors read and approved the final manuscript.

## Figures and Tables

**Table 1:** Furosemide regimen

Furosemide	Before	0–24 hours	24–48 hours	48–72 hours
Furosemide bolus IV				
Patients (*n*)	7	4	2	1
Dose (mg/kg per 24 hours)	1.0 (0.4–2.4)	1.1 (1–3.6)	3.3 (1–3.6)	3.6
Bumetanide bolus IV				
Patients (*n*)			1	
Dose (mg/kg per 24 hours)			0.1	
Continuous IV furosemide				
Patients (*n*)		31	25	23
Dose (mg/kg per hour)		0.08 (0.02–0.17)	0.08 (0.02–0.17)	0.08 (0.02–0.17)
Total IV furosemide				
Patients (*n*)		31	25	23
Dose (mg/kg per 24 hours)		1.92 (0.96–6.6)	1.92 (0.48–6.6)	2.0 (0.5–6.6)

Data are presented as median (range). IV, intravenous.

**Table 2:** Furosemide trials

Furosemide	Singh [5] prospective RCT 24 hours (1992)	Luciano [6] prospective RCT 24 hours (1997)	Klinge [7] prospective RCT 72 hours (1997)			van der Vorst [8] prospective observational 72 hours (2001)		
Intermittent								
Patients (*n*)	12	15	23					
Continuous								
Patients (*n*)	8	11	23			12		
Intermittent								
Age	1.44 (± 1.4) years	3.7 (± 3.4) months	2.4 (± 2.1) years			13 (0–33) weeks^a^		
Continuous								
Age	2.3 (± 2.2) years	1.8 (± 2.5) months	3.4 (± 3.1) years					
*P*value	NS	0.1	NS					
Study day			1	2	3	1	2	3
Intermittent dose mg/kg per 24 hours	6.23 (± 0.62)	6.8 (± 1.2)	1.6 (± 0.6)	0.9 (± 0.5)	1.0 (± 0.5)			
Continuous dose mg/kg per 24 hours						2.2 (± 0.4)	4.2 (± 1.1)	3.6 (± 1.3)
*P *value	0.045	0.001	0.014	0.0003	0.014			
Intermittent UO (ml/kg per hour)	3.53 (± 4.1)	3.3 (± 1.1)	3.1 (± 0.8)	2.9 (± 1.1)	2.9 (± 1.0)			
Continuous UO (ml/kg per hour)	3.36 (± 1.79)	2.5 (± 1.1)	2.7 (± 0.8)	2.9 (± 0.9)	3.6 (± 1.1)	2.4 (0.6–5.2)^a^	5.8 (3.5–9.1)^a^	5.4 (3.6–7.4)^a^
*P *value	NS	0.05	NS	NS	NS			
Intermittent UO/variance	13.07 (± 14.56) ml/kg per hour	3.8 (± 2.1)						
Intermittent UO/variance maximal			15.8 (± 3.7) ml/kg per hour					
Intermittent UO/variance minimal			0.3 (± 0.2) ml/kg per hour					
Continuous UO/variance	2.19 (± 1.92) ml/kg per hour	1.9 (± 1.6)						
Continuous UO/variance maximal			9.4 (± 4.1) ml/kg per hour					
Continuous UO/variance minimal			0.5 (± 0.3) ml/kg per hour					
*P *value	0.045	0.02	< 0.0001					

^a^Median (range). Data given as mean (standard deviation) unless indicated otherwise. NS, not significant; RCT, randomised controlled trial; UO, urine output.
